# LIM-HD transcription factors control axial patterning and specify distinct neuronal and intestinal cell identities in planarians

**DOI:** 10.1098/rsob.230327

**Published:** 2023-12-13

**Authors:** M. Dolores Molina, Dema Abduljabbar, Anna Guixeras, Susanna Fraguas, Francesc Cebrià

**Affiliations:** ^1^ Department of Genetics, Microbiology and Statistics, Faculty of Biology, University of Barcelona, Barcelona, Spain; ^2^ Institute of Biomedicine of the University of Barcelona (IBUB), Barcelona, Spain

**Keywords:** planaria, regeneration, neoblast, LIM homeodomain, *lhx*, nervous system

## Abstract

Adult planarians can regenerate the gut, eyes and even a functional brain. Proper identity and patterning of the newly formed structures require signals that guide and commit their adult stem cells. During embryogenesis, LIM-homeodomain (LIM-HD) transcription factors act in a combinatorial ‘LIM code’ to control cell fate determination and differentiation. However, our understanding about the role these genes play during regeneration and homeostasis is limited. Here, we report the full repertoire of LIM-HD genes in *Schmidtea mediterranea*. We found that *lim homeobox* (*lhx*) genes appear expressed in complementary patterns along the cephalic ganglia and digestive system of the planarian, with some of them being co-expressed in the same cell types. We have identified that *Smed-islet1, -lhx1/5-1, -lhx2/9-3, -lhx6/8, -lmx1a/b-2* and -*lmx1a/b-3* are essential to pattern and size the planarian brain as well as for correct regeneration of specific subpopulations of dopaminergic, serotonergic, GABAergic and cholinergic neurons, while *Smed-lhx1/5.2* and *-lhx2/9.1* are required for the proper expression of intestinal cell type markers, specifically the goblet subtype. LIM-HD are also involved in controlling axonal pathfinding (*lhx6/8*), axial patterning (*islet1*, *lhx1/5-1, lmx1a/b-3*), head/body proportions (*islet2*) and stem cell proliferation (*lhx3/4, lhx2/9-3, lmx1a/b-2, lmx1a/b-3*). Altogether, our results suggest that planarians might present a combinatorial LIM code that controls axial patterning and axonal growing and specifies distinct neuronal and intestinal cell identities.

## Graphical abstract

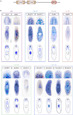

## 1. Introduction

Planarians can regenerate damaged or missing tissues, organs or entire organisms to full function within a period of a few days to several weeks. The extraordinary regenerative capacity of planarian relies on the presence of a population of adult stem cells named neoblasts [1–4]. How neoblasts achieve their final differentiation into the multiple cell lineages remains to be fully answered, although important progresses have been made in recent years (reviewed in [5]). In any case a combination of intrinsic and extrinsic signals and stimuli emanating from differentiated tissues and organs play a role in regulating neoblast biology [6–10] and need to be tightly coordinated and controlled for a successful regeneration.

Transcription factors (TFs) play key roles in multiple aspects of animal development. In planarians, many conserved TFs have been identified [11] and characterized to be required for the regeneration of multiple cells, tissues and organs including the intestine [12–15], the eyes [16–19], the central nervous system [20–28], the epidermis [29,30], the pharynx [31], the pigment cells [32,33], the musculature [34,35], the excretory system [36], as well as for establishing axial polarity [37–44].

LIM-homeodomain (LIM-HD) proteins are a family of transcription factors that play a crucial role in cell fate specification, differentiation and migration during embryonic development, especially for neural fates (reviewed in [45–47]). Structurally, LIM-HD proteins are characterized by the presence of two LIM domains, which mediate protein–protein interactions, and the homeodomain, which binds to specific DNA sequences and regulates gene expression (reviewed in [46]). LIM domains and homeodomains are found in non-metazoan eukaryotes, but the specific combination of LIM-LIM-HD is only found in metazoa [48]. During development of both vertebrate and invertebrate organisms, the expression of different combinations of *lim homeobox* (*lhx*) genes are considered to form a ‘LIM code’ that aids in specification of neural types within a tissue or organ and guides the establishment of topographically arranged connections [45,49–51]. For instance, during embryonic development of the mouse embryo, Lhx6 and Lhx7 specify GABAergic and cholinergic fates in cortical and forebrain neurons, respectively [52], while the axonal patterns, synaptic targets and neurotransmitter profiles of dorsal hindbrain interneurons are instructed by Lhx1/5, Lmx1b and Lhx2/9 [53]. Moreover, LIM-HD have been involved in endodermal specification [54–56], blastopore organizer activity [57], head formation [58] and in the regulation of the proliferation and migration of progenitor cells [59]. Although the important role of LIM-HD transcription factors during embryonic development has been well documented, our understanding about the role these genes may play during regeneration is limited.

Previous studies have started to study the function of LIM-HD during planarian regeneration [14,23,27,28,37,43,60,61]. Here, we report the identification of the full LIM-HD repertoire in *Schmidtea mediterranea* that includes thirteen homologues belonging to the six evolutionary conserved types of *lhx* genes. We found that ten of them are expressed in the central nervous system (CNS) and two with intestinal cells. A systematic functional RNAi analysis has uncovered Smed-LIM-HD functions in the specification of neural and intestinal cellular subtypes. We report that *Smed-islet1, -lhx1/5-1, -lhx2/9-3, -lhx6/8, -lmx1a/b-2* and -*lmx1a/b-3* are essential to pattern and size the planarian brain as well as for the correct specification of subpopulations of dopaminergic, serotonergic, GABAergic and cholinergic neurons. *Smed-lhx1/5.2* and *Smed-lhx2/9.1* are required for the proper expression of diverse intestinal cell type markers, especially of the goblet subtype. Other LIM-HD are also involved in the control of axonal pathfinding (*lhx6/8*), axial patterning (*islet1*, *lhx1/5-1*and *lmx1a/b-3*), head/body proportions (*islet2*) and stem cell proliferation (*lhx3/4, lhx2/9-3, lmx1a/b-2* and *lmx1a/b-3*) in planarians. Altogether, our results suggest that planarian LIM-HD could provide a combinatorial LIM code to specify distinct neuronal and intestinal cell identities as well as to control axial patterning and axonal growing.

## 2. Results

### 2.1. Thirteen *lim homeobox* genes are present in *Schmidtea mediterranea*

**Figure 1.**
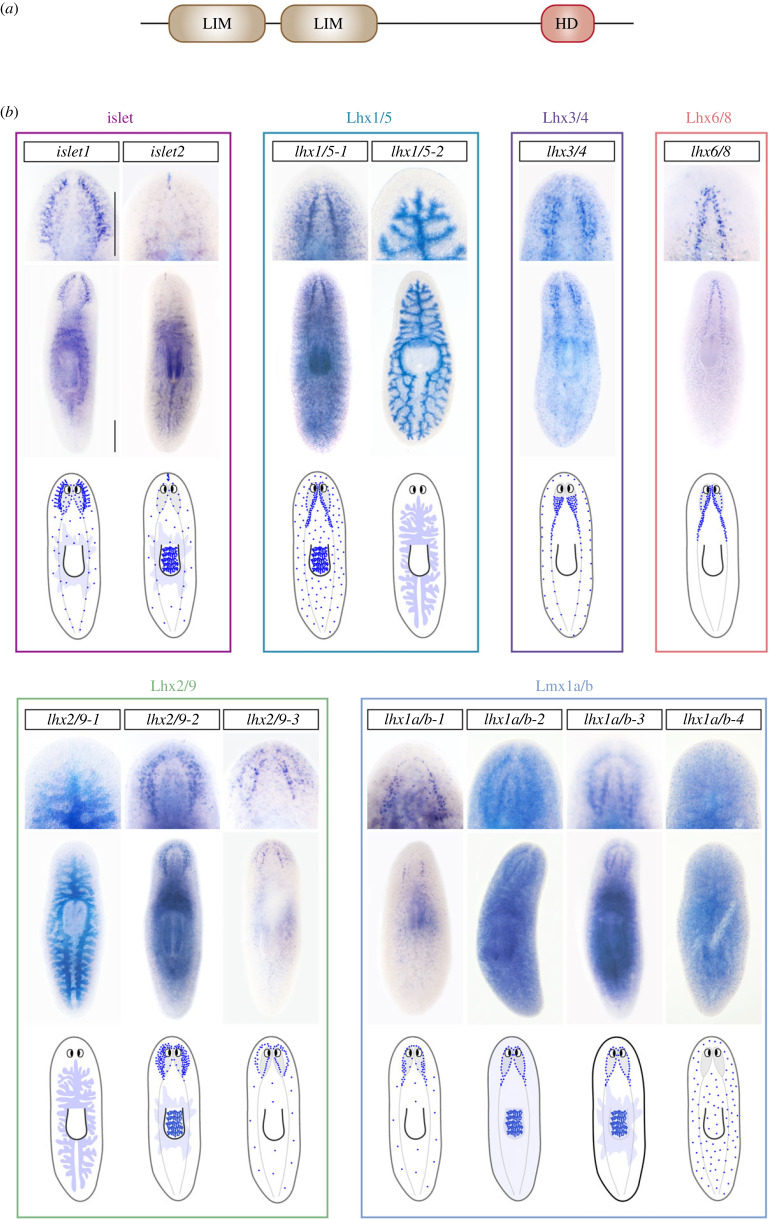
Planarian *lhx* are expressed in diverse domains of the cephalic ganglia and the digestive system. (*a*) Schematic showing the domain arrangement of the *S. mediterranea* LHX proteins. The distinct domains are highlighted in different colours. (*b*) Representative expression patterns of planarian *lhx* genes in intact planarians as determined by whole mount *in situ* hybridization. The anterior end of the body is oriented towards the top. A magnification of the ventral view of the cephalic region or the dorsal view of the digestive system is shown in the upper panel for each of the genes analysed. Scale bars: 400 µm.

Previous work had characterized 5 homologues of the LIM-HD family in planarians [14,23,27,28, 37,43,60,61]. By *in silico* searches, we identified the presence of 13 *lhx* genes in the genome of *Schmidtea mediterranea* (as also reported recently [62]). All identified genes code for LIM-HD proteins that present the characteristic pair of LIM domains at the N-terminal and a Homeodomain at the C-terminal region (figure 1*a*). Phylogenetic analysis indicated that planarians possess representatives of all six major LIM-HD subfamilies (electronic supplementary material, figure S1). In particular, *S. mediterranea* possesses two *islet* genes, *Smed-islet1* [28,43] and *Smed-islet-2* [60], two *lhx1/5* genes, named *Smed-lhx1/5-1* [27] and *Smed-lhx1/5-1-2*, three members of the lhx2/9 gene subfamily, *Smed-lhx2/9-1* [14]; *Smed-lhx2/9-2*; *Smed-lhx2/9-3*, a member for each of the *lhx3/4* and *lhx6/8* subfamilies, *Smed-lhx3/4* and *Smed-lhx6/8* [23], respectively, and four homologues of the *lmx1a/b* subfamily, that were named *Smed-lmx1a/b-1*, *Smed-lmx1a/b-2*, *Smed-lmx1a/b-3*, *Smed-lmx1a/b-4*.

### 2.2. *Lim homeobox* genes are mainly expressed in the planarian brain and digestive system

Having identified the existing LIM-HD genes in planaria, we sought to determine their expression patterns via *in situ* hybridization in intact (figure 1*b*) and regenerating (electronic supplementary material, figure S2) planarians as well as by analysing available single-cell (SC; electronic supplementary material, figure S3) and bulk transcriptomic data (electronic supplementary material, figures S3, S4).

Two planarian *lhx* genes, *lhx1/5-2* (this work) and *lhx2/9-1* [14], appeared to be strongly expressed in the planarian gut (figure 1*b*; electronic supplementary material, figure S2). The planarian intestine is a highly branched organ that consists of one anterior and two posterior primary gut branches that project into the head and tail, respectively, and connect to a centrally located pharynx which evaginates ventrally through the mouth opening for feeding. Three distinct cell types have been reported within the planarian intestine: absorptive phagocytic cells, secretory goblet cells and basal cells, which are located in proximity to the basal region of the phagocytes [14,63]. According to available single cell data, *lhx1/5-2* is mainly expressed in differentiated phagocytes and in gut progenitor cells, while the expression of *lhx2/9-1* is concentrated within differentiated basal and goblet cell types (electronic supplementary material, figure S3) [14,63,64].

Notably, the expression of 10 out of the 13 planarian *lim homeobox* genes was concentrated in the central nervous system (CNS) (figure 1*b*; electronic supplementary material, figure S2). The CNS of the planaria consists of a pair of cephalic ganglia that are located in the anterior region of the head as well as a pair of ventral nerve cords that run all along the anterior–posterior axis of the animal [65–67]. Several studies have shown that the planarian brain is a complex organ that contains a large number of neural subtypes and that are regionalized into domains of gene expression along the anterior–posterior, dorsal–ventral and medio-lateral axes [66,68,69]. Interestingly, we identified that several *lhx* genes were expressed in distinct domains within the planarian brain (figure 1*b*; electronic supplementary material, figure S2). We confirmed previous studies that reported *Smed-islet1* expression in the anterior and posterior poles during the early stages of regeneration (electronic supplementary material, figure S2) [28,43]. Additionally, we observed strong expression of *Smed-islet1* in the planarian brain, particularly in the lateral brain branches, as well as in the parapharyngeal region of the body of intact animals (figure 1*b*). SC data confirmed the expression of *Smed-islet1* in differentiating secretory, cholinergic (*chat+*) and GABAergic neurons as well as in progenitor neural cells (electronic supplementary material, figure S3). We could also confirm the expression of *Smed-islet2* in the most anterior tip of the planarian head (figure 1 and electronic supplementary material, figure S2) [60]. In addition, we detected few scattered *islet2* expressing cells around the cephalic ganglia and throughout the planarian body as well as a broad domain of *islet2* expression in the pharynx and parapharyngeal region. These *islet2* expressing cells could correspond to differentiated secretory, cholinergic neurons (*chat+*) and epidermal cells according to SC data (electronic supplementary material, figure S3). In agreement with previous reports, our studies detected *Smed-lhx1/5-1* expression in a large number of discrete neural cells distributed throughout the body and that appeared particularly dense in the medial domain of the cephalic ganglia (figure 1 and electronic supplementary material, figure S2) [27]. Previous reports have also shown *lhx1/5-1* expression in stem cells [27]. In addition, we observed strong *lhx1/5-1* expression in the pharynx, both by *in situ* and in SC transcriptomic data (figure 1 and electronic supplementary material, S3). Finally, the already reported expression of *Smed-lhx6/8* (arrowhead) was also confirmed and detected in discrete cells that locate medially within the planarian brain [23] and that could correspond to differentiated cholinergic and GABAergic neurons according to SC information (figure 1*b*; electronic supplementary material, figures S2 and S3).

The expression patterns of previously uncharacterized *lhx* genes were also studied in detail. The expression of the single *lhx3/4* homologue was detected mainly in discrete neurons that appear predominantly concentrated in the most posterior region of the brain lobes, as well as in discrete cells located along the nerve cords and the planarian body edges (figure 1*b* and electronic supplementary material, figure S2). According to SC transcriptomic data these cells correspond to diverse differentiated cell types such as cholinergic and GABAergic neurons, muscular and parenchymal cells as well as to neural progenitor cells (electronic supplementary material, figure S3) [37,64]. Two out of the three *lhx2/9* genes present in planarians also appeared expressed in the cephalic ganglia. Strong *lhx2/9-2* expression was detected in the brain, particularly in an external domain that could relate to the lateral brain branches, as well as in the parapharyngeal body region (figure 1*b* and electronic supplementary material, figure S2). According to SC transcriptomic data *lhx2/9-2* expressing cells relate to differentiated secretory, pharyngeal, muscular, and neural (*chat*+) cells, as well as to progenitors for the muscular and epidermal lineages (electronic supplementary material, figure S3). Similarly, the expression of *lhx2/9-3* was particularly enriched in the external domain of the cephalic ganglia that could relate to the lateral brain branches. We also found some scattered *lhx2/9-3* expressing cells distributed throughout the planarian body (figure 1*b* and electronic supplementary material, figure S2). According to SC information, these *lhx2/9-3* cells could correspond to differentiated muscular and neural (*chat+*, GABAergic) cells as well as to muscular and neural progenitor cells (electronic supplementary material, figure S3).

Finally, the expression of the four genes of the LMX1a/b subfamily was also characterized. Whole-mount *in situ* hybridizations revealed *lmx1a/b-1* expression in a discrete row of few neurons that seemed to trace the most anterior region of the brain commissure. Disperse *lmx1a/b-1* expressing cells were also found throughout the planarian body. Some of them appeared particularly condensed at the most posterior domain of the cephalic region. Those *lmx1a/b-1* expressing cells located ventral to the cephalic ganglia and might be defining a subdomain within the ventral nerve cords (figure 1*b* and electronic supplementary material, figure S2). In contrast to *lmx1a/b-1*, the expression of *lmx1a/b-2* and *lmx1a/b-3* appeared uniform and extended throughout the cephalic ganglia and the planarian body (figure 1*b* and electronic supplementary material, figure S2). *lmx1a/b-2* expression appeared relatively ubiquitous and SC transcriptomic data suggested that *lmx1a/b-2* transcripts are detected in differentiating neurons (*otf+, npp18+*) as well as in neoblasts and progenitor cells for the epidermal, muscular, intestinal and neural lineages, while *lmx1a/b-3* expression is also detected in several cell types, but mainly in differentiated protonephridia and neurons (*otf*+ *GABAergic*, *chat+*), as well as in epidermal, muscular, and neural progenitor cells (electronic supplementary material, figure S3). Finally, *Smed-lmx1a/b-4* expression was detected in discrete cells distributed all along the dorsal and ventral body. Available SC data suggest that *lmx1a/b-4* positive cells possess secretory (electronic supplementary material, figure S3) [64] and/or neural identity [63].

Our analyses reveal that *lhx* genes were broadly expressed in the planarian body, particularly in the neural, intestinal, pharyngeal and muscular lineages, as well as in several stem cell progenitor subtypes. In general, during anterior regeneration, all *lhx* genes were expressed within the growing blastemas since early after amputation (electronic supplementary material, figures S2, S4). In posterior regeneration, however, the expression of *lhx6/8*, *lmx1a/b-1* and *islet 2* was very low (electronic supplementary material, figures S2, S4). When analysing the response of planarian *lhx* genes to wounding within the first twelve hours, they clustered into two distinct groups, with five genes showing very low upregulation during this period and eight genes showing a strong upregulation since very early after wounding, especially *lmx1a/b-2* and *lhx1/5-2* (electronic supplementary material, figure S4)*.* The expression patterns observed for several *lhx* in subdomains along the anterior–posterior (*lhx3/4*) and medio-lateral (*islet1*, *lhx1/5-1*, *lhx6/8*, *lhx2/9-2* and *lhx2/9-3*) axes of the cephalic ganglia further evidence the molecular complexity of the planarian brain. Moreover, our analysis of available SC data identified a number of planarian cells co-expressing a combination of two different *lhx* genes (electronic supplementary material, figure S5). In agreement, we could confirm by double *in situ* hybridization that some planarian *lhx* appeared co-expressed in cells around the cephalic ganglia, presumably neurons. In particular, we identified co-expression of *lhx2/9-3* and *islet1*, as well as of *lhx2/9-3* and *lhx2/9-2* genes but not of *lhx2/9-3* and *lmx1a/b-1* transcripts. Interestingly, we observed that all cells positive for *lhx2/9-3* did also express *lhx2/9-2*, but this *lhx2/9-2* expression seemed lower when compared to the staining observed in *lhx2/9-2^+^/lhx2/9-3^−^* cells (electronic supplementary material, figure S5). Altogether, our data suggest that a ‘LIM code’ of *lhx* gene expression could be present in planarians to aid in the specification of diverse neural types and/or guide in establishing topographically arranged neural connections.

### 2.3. Planarian *lhx* genes are needed for proper visual axonal projections and brain regeneration

**Figure 2.**
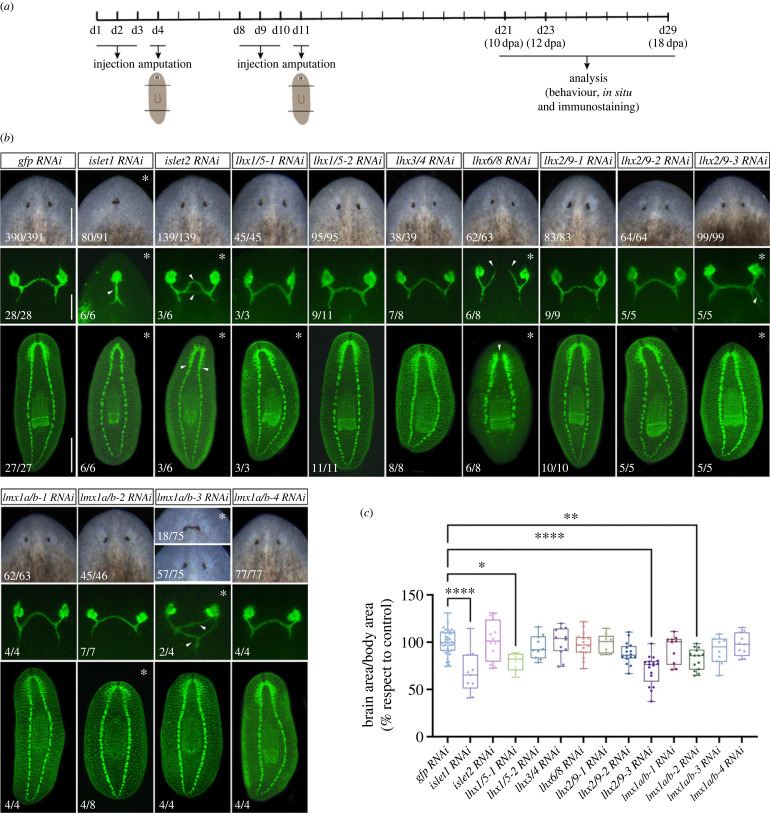
Planarian LIM-HD are required for proper visual axonal projections and brain regeneration. (*a*) Schematic representation of the dsRNA injection experimental procedure. (*b*) Defects in RNAi treated planarians: upper panels show live images of anterior facing regenerated blastemas (scale bar: 150 µm). Middle panels show regenerated eye photoreceptor cells and their axonal projections as labelled with the VC1 antibody (scale bar: 150 µm). Bottom panels show the planarian CNS as labelled with the anti-SYNAPSIN antibody (scale bar: 500 µm). All animals correspond to trunk pieces at 10–12 days of regeneration after 2 rounds of RNA interference. Asterisks highlight the panels where defects in the regenerative process are observed. White arrowheads in middle panels for *islet1; -2; lhx6/8; lhx2/9-3;* and *lmx1a/b-3* (RNAi) planarians point to aberrant eye photoreceptor axonal projections. White arrowheads in bottom panels point to brain ganglia that elongate posteriorly in *islet2*(RNAi) planarians and to a medial gap in the brain commissure in *lhx6/8*(RNAi) animals. (*c*) Graphical representation of the ratio of the brain-to-body area in control *gfp*(*RNAi*) and after silencing planarian *lhx* genes. The brain and body area of planarians at 10–12 days of anterior and posterior regeneration were analysed. Between 6 and 40 animals were analysed per RNAi condition. Values in graphs are represented as % with respect to the mean of control *gfp*(RNAi) animals (*p-value < 0.05; **p-value < 0.01; **** *p*-value < 0.0001).

To characterize the function of *Smed-lhx* genes we performed RNA interference (RNAi)-based functional analyses. Animals were amputated pre- and post-pharyngeally after dsRNA delivery and the regenerative process monitored for 10–18 days (figure 2). A summary table of the phenotypes observed can be found in electronic supplementary material, file S1.

Apparent normal regeneration of the anterior blastemas was observed after silencing most of the planarian *lhx* genes (figure 2*b*). However, the silencing of two *lhx* genes impaired anterior regeneration. Thus, RNAi against *islet1* and *lmx1a/b-3* resulted in midline defects and regeneration of merged eyes in *n* = 80/91 and *n* = 18/75 of the analysed animals, respectively. Our results for the silencing of *islet1* agree with previous reports [28,43]. Also, in most conditions no obvious morphological defects were observed during posterior regeneration with the exception of the rounded and smaller blastemas observed in all *islet1* silenced animals (data not shown, see next sections).

As we found that most of the *lhx* genes were expressed in the CNS of the planarian, we investigated whether they were needed for the proper regeneration of the visual axons and the CNS. To do that we performed immunostainings against eye photoreceptor cells and with a panneural antibody. Control *gfp*(RNAi) planarians regenerated eyes and visual axonal projections that connected stereotypically to the brain forming proper optic chiasms (figure 2*b*) [70]. We observed that abnormal visual axonal projections towards the planarian brain accompanied the midline defects and cyclopic eyes observed in silenced *islet1* and *lmx1a/b-3* animals (figure 2*b*). Remarkably, axonal growth of the photoreceptor cells was also found perturbed in the apparently normal regenerated eyes of *islet2*, *lhx6/8*, *lhx2/9-3* and *lmx1a/b-3* silenced planarians, suggesting a role for those genes in axonal growth pathfinding (asterisks, figure 2*b*). The most striking phenotype was observed after silencing *lhx6/8*. In those animals, the visual axonal projections completely failed to cross the midline and connect to the contralateral side. These results agree with previous data that reported a role of *lhx6/8* on defining neurons that serve as guidepost cells for de novo regeneration of the visual system [23,61].

Analyses of the nervous system of *lhx*(RNAi) animals visualized with the panneural marker anti-synapsin identified that the regeneration of the cephalic ganglia was altered after silencing several *lhx* genes (figure 2*b*). In particular, regeneration of fused or elongated brain ganglia occurred in *islet1* and *islet2* silenced planarians, respectively; moreover, RNAi of either *islet1*, *lhx1/5-1*, *lhx2/9-3* or *lmx1a/b-2* resulted in significantly smaller brains (figure 2*c*). Finally, the silencing of *lhx6/8* disrupted the medial anterior commissure that connects both cephalic ganglia, in agreement of the observed visual axonal defects and previous reports [23,61].

Planarians show a negative phototactic behaviour as they move away from light [71]. To investigate whether the defects in the regeneration of the visual system and the CNS observed after silencing *lhx* genes correlated with abnormal movement and negative phototaxis we exposed planarians to a light gradient and recorded and analysed their response. Control *gfp*(RNAi) planarians reacted to light and moved away to reach the shallowed region of the container (electronic supplementary material, figure S6 and video S1). This normal behaviour was also observed in planarians silenced for *islet2*, *lhx1/5-2*, *lhx2/9-1*, *lhx2/9-2, lhx2/9-3*, *lhx6/8*, *lmx1a/b-2* or *lmx1a/b-3* (electronic supplementary material, figure S6 and videos S3, S5–S11). By contrast, few *islet1* and most *lhx1/5-1* silenced animals displayed reduced mobility and negative phototactic response and did not reach the shallowed region in the time of the experiment (electronic supplementary material, figure S6 and videos S2, S4). Most *lhx1/5-1* animals also exhibited abnormal gliding behaviour (as already reported by [27]) and reduced ability to flip back onto the dorsal surface (electronic supplementary material, video S4).

Finally, we investigated the proliferative rate of stem cells and carried out immunostaining against a phosphorylated form of Histone-3 that identifies the G2/M stage of the cell cycle. We quantified the total number of mitoses in bilateral amputated planarians regenerating anterior and posterior wounds at 10 days after amputation. In agreement with the normal blastema formation observed in most *lhx* silenced planarians, we did not observe a decrease in the proliferative rate of stem cells. On the contrary, in four of the RNAi conditions (*lhx3/4, lhx2/9-3, lmx1a/b-2* and *lmx1a/b-3)* we observed significantly increased rates of neoblast proliferation when compared to control *gfp*(RNAi) planarians (electronic supplementary material, figure S7). Notably, all these four *lhx* genes were expressed in planarian stem cells (electronic supplementary material, figure S3) [37], suggesting that they could function autonomously in regulating planarian stem cell proliferative rates.

### 2.4. Planarian *lhx* genes, especially *islet1*, are needed for correct lateral regeneration and midline patterning

**Figure 3.**
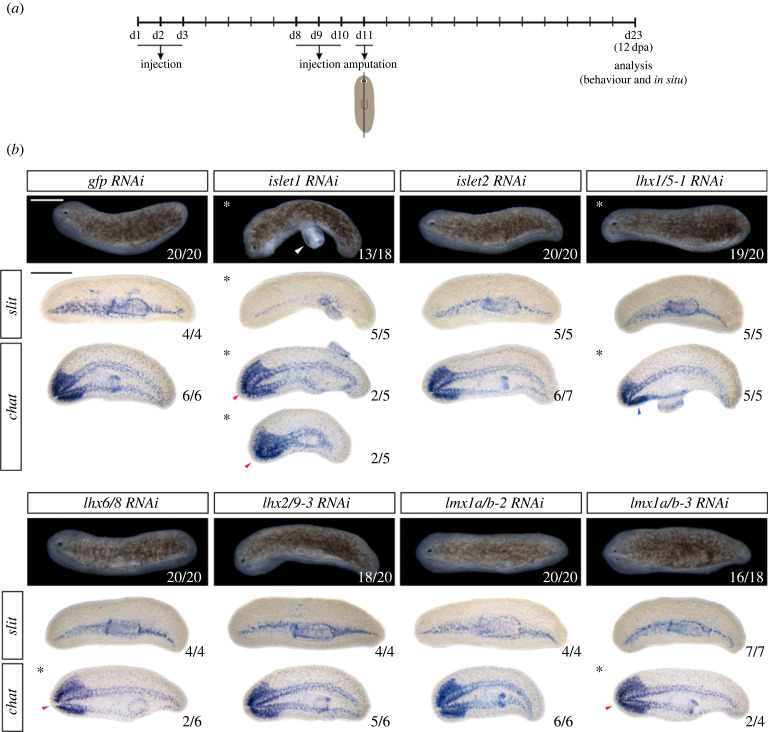
Perturbed lateral regeneration after *islet1* and *lhx1/5-1* silencing. (*a*) Schematic representation of the dsRNA injection experimental procedure. (*b*) Defects in RNAi treated planarians: upper panels in each RNAi condition show live images of planarians regenerating laterally after *lhx* silencing. Bottom panels show expression of the midline marker *slit* and the neural marker *chat* in *lhx* silenced planarians. All animals correspond to 12 days of regeneration after 2 rounds of RNA interference. Asterisks highlight the panels where defects in the regenerative process are observed. White arrowhead in the *islet1* panel points to an evaginated pharynx. Red arrowheads in panels for *islet1*, *lhx6/8* and *lmx1a/b-3* (RNAi) planarians point to aberrant brain commissures. Blue arrowhead in the *lhx1/5-1* panel points to the small, regenerated brain ganglia. Scale bar: 100 µm.

To further investigate the patterning and midline defects observed in silenced *islet1*, *islet2*, *lhx1/5-1*, *lhx6/8*, *lhx2/9-3*, *lmx1a/b-2* and *lmx1a/b-3* planarians we analysed their behaviour and ability to regenerate laterally after sagittal amputation (figure 3). Most RNAi-treated planarians regenerated normal lateral blastemas after sagittal amputation, except some animals that regenerated smaller blastemas (silencing of *islet1* or *lhx1/5-1*) and brains (arrowhead in *lhx1/5-1* silenced animals) or displayed aberrant brain commissures (red arrowheads after *islet1*, *lhx6/8* or *lmx1a/b-3* silencing) compared to control *gfp*(RNAi) planarians (figure 3*b*). Similar to anterior–posterior regeneration, the negative phototactic response of some lateral regenerating planarians was perturbed after RNAi treatment. In particular, the majority of *islet1, lhx1/5-1* and *lhx6/8* silenced planarians showed reduced mobility and limited negative phototactic behaviour compared to control *gfp*(RNAi) animals, while only a slight delay in the negative response to light was observed in few animals silenced for *islet2*, *lhx2/9-3*, *lmx1a/b-2* or *lmx1a/b-3* (electronic supplementary material, figure S8 and videos S12–S19).

Midline patterning defects were mainly observed in *islet1* silenced animals. These animals failed to regenerate bilateral brains (red arrowhead in *islet1*) and a new eye. The expression of *slit*, a gene essential for regeneration and maintenance of the planarian midline whose silencing results in similar defects [72], was strongly reduced in those *islet1* RNAi regenerating animals, suggesting that their midline defects could be caused by insufficient *slit* expression. In agreement, previous work identified the requirement of *islet1* for expression of *slit* at the midline of anterior and posterior blastemas at early stages of regeneration [28]. Our data suggest that *islet1* is also required for strong expression of *slit* all along the planarian midline during lateral regeneration. In addition to perturbed regeneration and midline patterning defects, we observed an uncommon behaviour in *islet1* silenced animals regenerating laterally as the pharynx of these animals was constantly evaginated and appeared extruded (white arrowhead in figure 3*b*). A similar behaviour was observed in bipolar regenerating *islet1*(RNAi) planarians in the presence of food. Compared to control *gfp*(RNAi) treated planarians, the feeding behaviour of *islet1* silenced animals regenerated anterior–posteriorly was compromised as the animals extruded their pharynxes and move in circles instead of towards the food (electronic supplementary material, videos S20–S21).

### 2.5. LIM-HDs specify distinct neural subtypes and are needed for correct patterning of the planarian brain

**Figure 4.**
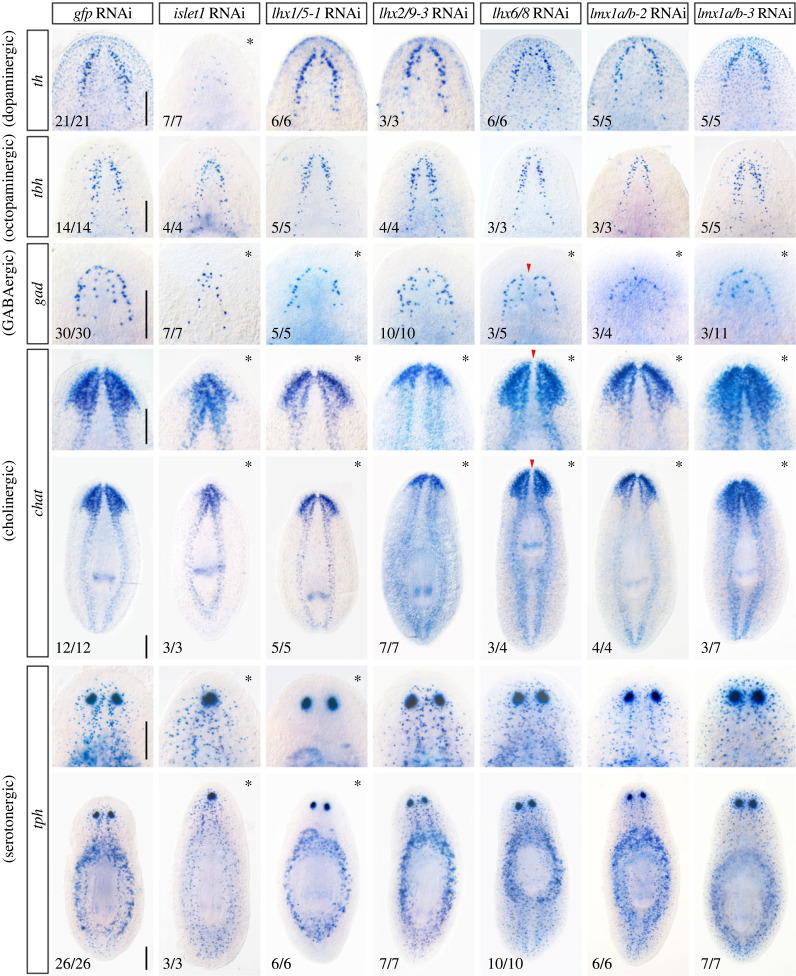
Aberrant regeneration of different neural cell subtypes after silencing planarian *lhx* genes. Whole mount *in situ* hybridizations for *th* (dopaminergic), *tbh* (octopaminergic), *gad* (GABAergic), *chat* (cholinergic) and *tph* (serotonergic) neural cell types in control *gfp*(RNAi) and after silencing of *lhx2/9-3, lhx6/8, lmx1a/b-2, lmx1a/b-3, lhx1/5-1* or *islet1* genes. All animals correspond to trunk pieces at 10–12 days of anterior–posterior regeneration after 2 rounds of RNA interference. Arrowheads in *lhx6/8*(RNAi) animals point to a medial gap in the brain commissure. Arrowheads in *lmx1a/b-3*(RNAi) point to a thicker brain commissure. Asterisks highlight the panels where defects are detected. Anterior to the top. Scale bar: 250 µm.

Regeneration of a functional brain requires the specification of several neural subtypes that need to be properly patterned and integrated into a functional unit. We investigated next if planarian LIM-HD are involved in defining neural cell type identity by analysing the presence of five main neural subtypes after silencing each of the 13 *lhx* genes by RNAi (figure 4 and electronic supplementary material, figure S9). We characterized the presence of dopaminergic neurons expressing *tyrosine hydroxylase* (*th*) [73], octopaminergic neurons expressing *tyramine beta-hydroxylase* (*tbh*) [74], GABAergic neurons expressing *glutamine decarboxylase* (*gad*) [75], serotonergic neurons expressing tryptophan hydroxylase (*tph*) [76] and cholinergic neurons expressing *choline acetyltransferase* (*chat*) [77].

Silencing of either *islet1* or *lhx1/5-1* perturbed the expression of the serotonergic neuronal marker *tph*. Serotonergic *tph*+ neurons distributed along the planarian body were completely absent after silencing *lhx1/5-1* (as previously reported by [27]), while *tph* expressing cells that locate around the pharynx and in the eye appeared unaffected. Notably, in contrast, we observed that *islet1*(RNAi) treatment decreased exclusively the parapharyngeal domain of expression of *tph* and did not perturb either scattered serotonergic *tph* expressing cells or eye *tph+* expressing cells (figure 4). Moreover, the silencing of *islet1* caused a strong reduction in the number of cells expressing the dopaminergic marker *th*; this neural subpopulation remained mainly unaffected after silencing any of the other *lhx* genes analysed (figure 4).

Regeneration of octopaminergic neurons as analysed by the expression of *tbh* was not affected after RNAi of any of the *lhx* genes (figure 4). Also, regeneration of GABAergic and cholinergic neurons occurred in all analysed RNAis. However, several patterning defects as well as defective regeneration of some subdomains of expression for those neural subtypes were observed (figure 4). We confirmed that *lhx1/5-1*(RNAi) worms lacked the ventral subpopulation of GABAergic cells (corresponding to the internal row of gad+ cells as observed from a dorsal view) [27], as well as the midline defects in *lhx6/8* (RNAi) treated animals which regenerated with a gap between the lobes of the cephalic ganglia (figure 4) [23]. In addition, we observed that silencing of *lhx1/5-1* resulted in reduced *chat* expression in the internal domain of the brain lobes, suggesting a mediolateral defect of the brain and the presence of a reduced number of cholinergic neurons on the medial region of the cephalic ganglia (figure 4). GABAergic and cholinergic neuron regeneration was also perturbed after silencing *lmx1a/b-2* and *lmx1a/b-3*. Similar to *lhx1/5-1*(*RNAi*), silencing of *lmx1a/b-2* resulted in reduced *chat* expression in the internal domain of the brain lobes (figure 4). Also, in agreement with the midline defects observed during regeneration of the planarian eyes, *chat* and *gad* expression in the anterior region of the cephalic ganglia appeared fused and thicker in *lmx1a/b-3* silenced worms. Finally, visualization of *chat* expressing cholinergic neurons confirmed that *lhx2/9-3* silenced planarians regenerated smaller brains compared to control *gfp*(RNAi) planarians (figure 4).

Altogether, these data suggest that LIM-HD transcription factors are required for correct regeneration and patterning of the planarian brain as well as to define distinct neural identities, particularly for the serotonergic and the dopaminergic subtypes.

### 2.6. *lhx1/5-2* and *lhx2/9-1* silencing perturbs intestinal gene expression

We observed enriched expression of *lhx1/5-2* and *lhx2/9-1* in the planarian digestive system (figure 1*b*). As already mentioned, three main cell types constitute this highly branched organ: phagocytes, basal cells and goblet cells. Phagocytic cells can be identified by the expression of the *cathepsin La* marker (*ctsla*) [14]. Basal cells can be visualized by the expression of the *solute carrier-family transporters 22 member 6* (*slc22a6*) [14]. Finally, secretory goblet cells located in the lateral region of the intestine can be identified by the enriched expression of a Kunitz-type protease inhibitor (*Smed_v6_370*, from now on 370), while goblet cells located in the medial region of the intestine can be identified by the enriched expression of the metalloendopeptidase *cg7631* [14] as well as by the presence of the RAPUNZEL-1 protein [78].

**Figure 5.**
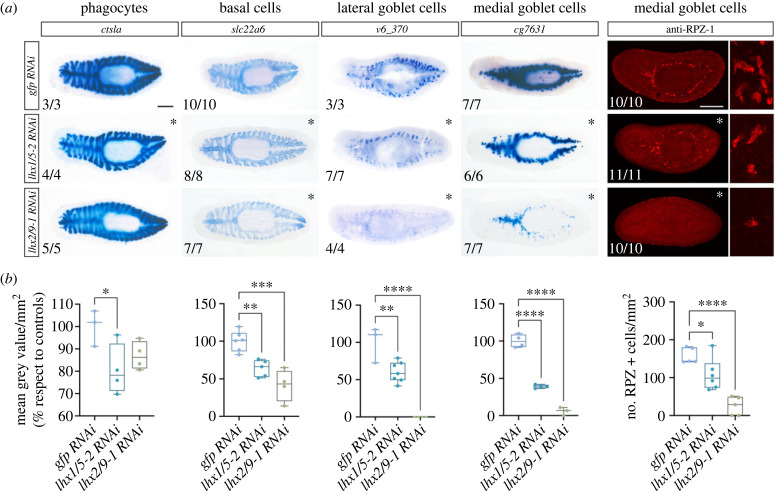
Defects in intestinal cell types after *Smed-lhx1/5-2* and *Smed-lhx2/9-1* silencing. (*a*) Whole mount *in situ* hybridizations for *ctsla* (phagocytes), *slc22a6* (basal cells), *v6_370* (lateral goblet), *cg7631* (medial goblet) and immunostaining for RPZ-1 (medial goblet) intestinal cell types in control *gfp*(RNAi) and after silencing of *lhx1/5-2 or lhx2/9-1* genes. All animals correspond to trunk pieces at 18 days of anterior–posterior regeneration after 2 rounds of RNA interference. Asterisks highlight the panels where defects are detected. Anterior to the left. (*b*) Graphical representation of the quantification of the signal intensity of the colorimetric staining for the intestinal markers analysed. Between 3 and 7 animals were analysed per RNAi condition. Values in graphs are represented as % with respect to the mean of control *gfp* RNAi animals, except for RPZ-1 where the values represent the quantification of the number of stained cells per body area. (*p-value < 0.05; **p-value < 0.01; *** *p*-value < 0.001; **** *p*-value < 0.0001). Scale bars: 250 µm.

We analysed the expression of *ctsla*, *slc22a6, 370* and *cg7631* after *lhx1/5-2* and *lhx2/9-1* RNAi treatment to decipher whether these genes play a role in gut regeneration and intestinal cell type specification and maintenance in planarians (figure 5). Silencing of either *lhx1/5-2* or *lhx2/9-1* caused defects in the expression of intestinal markers in the newly regenerated as well as the pre-existing intestinal tissue but did not seem to severely affect branching and regeneration of the new gut. We observed that silencing of *lhx1/5-2* significantly reduced the expression of the markers analysed for all three intestinal cell types, while *lhx2/9-1* silencing perturbed particularly the expression of markers for the basal and the lateral and medial goblet cell subtypes (figure 5*a*). Previous work reported a mild effect of *lhx2/9-1* silencing on goblet cell regeneration [14]. Our data revealed a much stronger effect, as the expression of the medial goblet cell marker *cg7631* was particularly reduced in both *lhx1/5-2* and *lhx2/9-1* silenced planarians and corresponded to about 39% and 3% of the expression observed in control *gfp*(RNAi) treated animals (figure 5*b*), while the expression of the lateral goblet cell marker *370* was practically absent in *lhx2/9-1* silenced planarians and was reduced to about 60% in *lhx1/5-2* silenced planarians compared to the expression observed in control animals. Similarly, quantification of RPZ-1 expressing cells confirmed the strong reduction in the number of medial goblet cells that silencing *lhx1/5-2* or *lhx2/9-1* genes caused (figure 5*b*). To investigate if these effects in gene expression correlated with disturbed feeding behaviour and intestinal function we fed RNAi treated planarians. Silencing of neither *lhx1/5-2* nor *lhx2/9-1* compromised planarian feeding behaviour (electronic supplementary material, figure S10 and videos S20, S22, S23) or viability, as such all animals ate and survived at least for three weeks after feeding.

These data indicate that the expression of *lhx1/5-2* and *lhx2/9-1* is required for the proper expression of intestinal cell markers as well as for the regeneration and maintenance of the goblet cells.

### 2.7. Islet1 and Islet2 are needed for correct patterning and body proportions

During planarian regeneration, the expression of genes specific to anterior or posterior wounds is required for the specification of head and tail identities as well as for the differentiation of proper anterior and posterior structures [1,79]. Early expression of the polarity genes *notum* and *wnt1* is stem-cell-independent and occurs after any injury, while late expression of *notum* and *wnt1* depends on the *proliferation* of the stem cells and locate to anterior and posterior wound sites, respectively [80,81].

**Figure 6.**
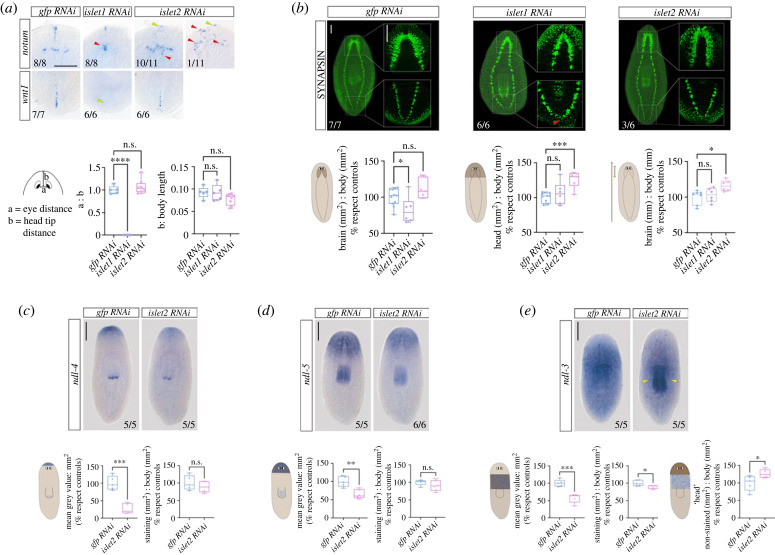
*islet1*(RNAi) and *islet2*(RNAi) defects in patterning and body proportions. (*a*) Whole mount *in situ* hybridizations for *notum* (in anterior blastemas) and *wnt1* (in posterior blastemas) genes in control *gfp*(RNAi) and after silencing *islet* genes. Red arrowheads point to aberrant expression patterns. Yellow arrowheads point to absent expression. All animals correspond to trunk pieces at 10 days of anterior–posterior regeneration after 2 rounds of RNA interference. Anterior to the top. Graphical representation of both the eye distance to head tip distance and the head tip distance with respect to the body length. Between 5 and 8 animals were analysed per RNAi condition. (**** *p*-value < 0.0001). Scale bar: 250 µm. (*b*) Immunostaining and confocal projections of the planarian CNS as labelled with the anti-synapsin antibody and graphical representation of the brain-to-body and the head-to-body ratios. Anterior and posterior confocal projections correspond to two different animals in the *islet2* RNAi condition. Red arrowhead points to fused posterior nerve cords. Values in graphs are represented as % with respect to the mean of control *gfp*(RNAi) animals. Between 6 and 11 animals were analysed per RNAi condition. (n.s., not significant; *p-value < 0.05; *** *p*-value < 0.0001). Scale bars: 250 µm. (*c–e*) Whole mount *in situ* hybridizations for *ndl-4* (*c*), *ndl-5* (*d*) and *ndl-3* (*e*) in control *gfp*(RNAi) and after silencing *islet2* and graphical representation of the quantification of the signal intensity and the area of the colorimetric staining for the markers analysed respect to the total body area. Yellow arrowhead in (*e*) points to aberrant expression of *ndl-3* in the pharynx in *islet2* silenced planarians. Anterior to the top. Values in graphs are represented as % with respect to the mean of control *gfp*(RNAi) animals. Between 5 and 6 animals at 13 days of anterior–posterior regeneration after 2 rounds of RNA interference were analysed per RNAi condition. Scale bars: 250 µm. (n.s., not significant; *p-value < 0.05; **p-value < 0.01; *** *p*-value < 0.0001).

Expression of *islet1* in anterior and posterior blastemas during early stages of regeneration has been reported to be important for the late and stem-cell-dependent expression of *wnt1* at posterior wounds and the establishment of posterior polarity [28,43]. As expected, we observed that *islet1* (RNAi) treated planarians lacked posterior identity and the late and localized expression of *wnt1* in posterior wounds (figure 6*a*), resulting in the regeneration of rounded posterior blastemas with fused nerve cords (figure 6*b*). In addition, we observed that the late and stem-cell-dependent expression of *notum* in anterior blastemas (figure 6*a*) merged at the midline after *islet1* silencing, as the regenerated eyes and cephalic ganglia also did in these planarians (figure 6*b*).

Our analysis of available SC databases identified cells co-expressing *islet2* and other anterior pole markers that are associated with the proper establishment of polarity, such as *foxD*, *zic*, *follistatin* or *notum* (electronic supplementary material, file S1) [39,81–84]. To clarify whether the expression of *islet2* in the anterior pole (figure 1) could also be associated with the establishment of polarity, we analysed the late and stem-cell-dependent expression of genes associated with anterior (*notum*) [81] and posterior (*wnt1*) [85] identity in *islet2*(RNAi) treated animals. *notum* and *wnt1* were found expressed and restricted to anterior and posterior blastemas, respectively, suggesting that anterior and posterior identities were specified in *islet2* silenced planarians (figure 6*a*). However, an unusual expression of *notum* was observed in the anterior blastema in *islet2*(RNAi) animals (figure 6*a*). The normal expression of *notum* in the most anterior tip of the head region was barely detected in *islet2* silenced planarians; moreover, the *notum* expressing cells associated to the anterior brain commissure and the eye photoreceptors [61,86] appeared more scattered and abnormally distributed in this anterior region after *islet2*(RNAi) (figure 6*a*).

The expression of *notum* in the head region has been associated with promoting brain size [86] and facilitating and guiding the regeneration of the planarian visual system [61]. Therefore, we analysed if the non-stereotypical pattern of *notum* expression observed in *islet2*(RNAi) planarians was linked to defects in brain size, eye positioning and/or visual axonal projections. As already mentioned, some aberrant projections of the visual axons toward the cephalic ganglia were observed in *islet2* silenced planarians (figure 2*a*). Eye distance and eye positioning along the anterior–posterior axis of the planarian body were found to be normal in those worms (figure 6*a*). Similarly, we found no differences in the size of the brain (figure 6*b*) neither in the number of *cintillo+* mechanosensory cells nor *gad+* GABAergic neurons (electronic supplementary material, figure S11), two population of neuronal cell types that have been strongly correlated with brain length and body size [86–88]. On the other hand, quantification of the head-to-body ratio revealed that head regions were larger in *islet2*(RNAi) planarians compared to control *gfp*(RNAi) animals (figure 6*b*). These results agree with the regeneration of normally sized but elongated brains after silencing *islet2* (figures 2*b*, 6*b*).

To study whether the change in brain shape was caused by general miss-patterning of the anterior region or was brain specific we analysed the expression of the *ndl-4* and *ndl-5* genes in the cephalic region [89]. The expression of these genes appeared strongly reduced in *islet2* silenced planarians, but their regionalized domains and areas of expression were similar in *islet2*(RNAi) and control organisms (figure 6*c*,*d*). Similarly, the intensity of expression of the *ndl-3* gene in the trunk medial region of the planarian, from below the eyes to the oesophagus at the anterior end of the pharynx, was strongly reduced in *islet2* silenced planarians. The area of expression of *nld-3* was smaller in *islet2*(RNAi) animals and located posterior along the anterior–posterior axis compared to control organism, further evidencing the larger head-to-body ratio of *islet2*(RNAi) planarians compared to control *gfp*(RNAi) animals (figure 6*b*,*e*). Finally, we detected a strong and ectopic expression of *ndl-3* in the pharynx of *islet2*(RNAi) treated animals (figure 6*e*).

Altogether, these data suggest that *islet2* is necessary for the stereotypical expression of *notum* in the head region, as well as for correct photoreceptor axonal projections, *ndl* gene expression and head-to-body allometric proportions.

## 3. Discussion

**Figure 7.**
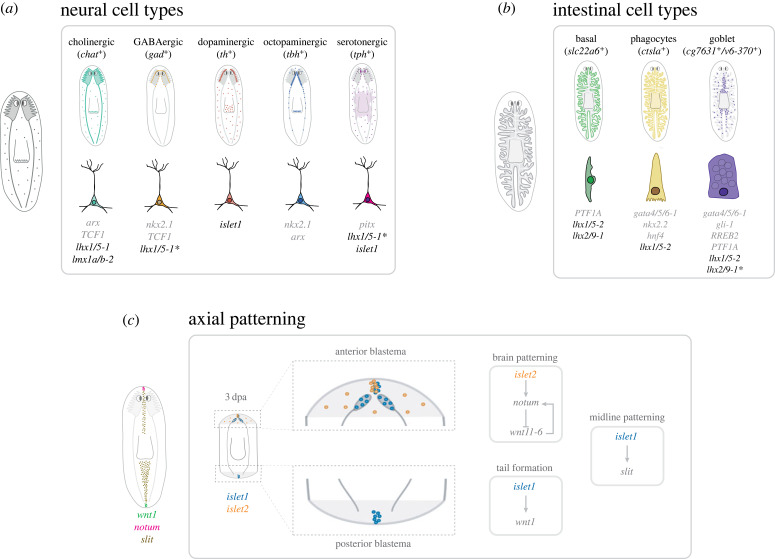
LIM-HD transcription factors specify distinct neuronal and intestinal cell type identities and control axial patterning in planarians. (*a*) Cartoon summarizing the role of planarian *lhx* genes and other previously characterized transcription factors in the specification of planarian cholinergic [21,90], GABAergic [21,27,90], dopaminergic, octopaminergic [90] and serotonergic [27] neural cell types. *, functions for Lhx1/5-1 already described [27]. (*b*) Cartoon summarizing the role of planarian *lhx* genes and other previously characterized transcription factors in the specification of basal [63], phagocytes [12–14,91] and goblet [12,14,63] intestinal cell types. *, function for Lhx2/9-1 already described [14]. (*c*) Scheme summarizing the role of planarian *islet* genes in axial patterning. *islet2* is expressed at the tip of the anterior blastema (orange cells) and is required for the expression of *notum* in the tip of the head as well as to create the stereotypical pattern of *notum*-expressing cells in the anterior commissure of the brain (see figure 6*a*). Our results on the function of Islet2 on brain patterning and head-to-body proportion could relate to the previously reported activity of *notum* and *wnt11-6* repressing neuron production and preventing posterior expansion of brain tissue [86]. *islet1* expression at the tip of the posterior blastema (blue cells) is required for the late, stem-cell-dependent phase of expression of *wnt1* and to maintain posteriorization (this work and [28,43]). Islet1 expression is also required for strong expression of *slit* and proper midline patterning (this work and [28]). 3 dpa, 3 days post-amputation.

In this work we provide functional evidence that LIM-HD genes are involved in specifying neuronal identity in planarians, especially the dopaminergic and serotonergic neuronal subtypes, as well as in controlling the expression of intestinal markers and the regeneration and maintenance of intestinal goblet cells (figure 7). Our data confirm the previously reported role for *lhx1/5-1* in maintaining serotonergic and GABAergic neural cells [27]. Also, our results suggest a role for *lhx1/5-1* and *lmx1a/b-2* in cholinergic neurons within the medial region of the planarian brain. In addition, we provide new evidence that *islet1* is necessary for the expression of *tph* in serotonergic parapharyngeal cells, as well as for the regeneration and maintenance of *th+* dopaminergic neurons. This lack of serotonergic parapharyngeal cells and dopaminergic neurons correlate with a compromised feeding behaviour and evaginated pharynges of *islet1* silenced animals suggesting that dopaminergic neuron signalling is needed for planarian feeding behaviour. In agreement, it has been shown that dopaminergic neurons signal the presence of food to interneurons that release neuropeptides and regulate locomotion in *C. elegans* [92].

In both vertebrates and invertebrates, neural subtypes are specified by combinatorial expression of LIM-HD and other transcription factors; however, it is important to point out that the same neurons are not generated by the same combinations in different species (reviewed in [45,46]). Previous studies in planarians have defined the role of several transcription factors in patterning the CNS and functionally specifying neural subtypes (figure 7*a*) (reviewed in [66]). Thus, for instance, Nkx2.1 and Arx are required for the maintenance of cholinergic (ventral medial), GABAergic (ventral medial), and octopaminergic (correct number) neurons [90]; TCF1 is required for the regeneration of dorsal GABAergic neurons [21], and Pitx and Lhx1/5-1 play a role in maintaining the identity and function of serotonergic neurons in the planarian CNS [27]. Here we report that Islet1 is required for maintenance and regeneration of dopamine neurons and parapharyngeal serotonergic cells. Thereby, our results suggest that in planarians there might also be a combinatorial activity of LIM-HD and other transcription factors to correctly specify and/or pattern several neural subtypes such as serotonergic, dopaminergic, GABAergic and cholinergic during maintenance and regeneration of the CNS (figure 7*a*).

In the developing mammalian limb, Lim1 and Lmx1b function in controlling the initial trajectory of motor axons and establishing the fidelity of a binary choice [93]. Similarly, in the fly, *lim3* participates in a combinatorial fashion with *tailup* (*Islet class*) to define the patterns of axonal projections of some motor neurons of the CNS [94]. Previous works have reported the role of Lhx6/8 in promoting reconnection of the brain lobes and the visual system during planarian regeneration [23,61]. Our data suggest that in addition to Lhx6/8, several other LIM-HD genes play a role in controlling the proper projections of the visual axons (Islet1, Islet2, Lhx2/9-3 and Lmx1a/b-3) and neural patterning (Islet1, Islet2, Lhx1/5-1, Lhx2/9-3, Lmx1a/b-2 and Lmx1a/b-3) during planarian regeneration.

In addition to the function of planarian LIM-HD in CNS patterning and neural specification, we report the role of two *lhx* genes (*lhx1/5-2* and *lhx2/9-1*) in the intestine. The current knowledge on the role of LIM-HD during intestinal development in other model systems is limited. It is known that Islet1 expression in the embryonic stomach activates Gata3 transcription to ensure normal pyloric development in the mouse [95]. Also, high levels of expression of Lhx1, Islet1, Islet2, and Lmx1a and the expression of Islet1 in stem-like cells have been reported in the mouse intestinal epithelium [96]. Lhx1 is also known for its role in blastoporal organizer activity during gastrulation [46], as well as endodermal specification in ascidians [54], amphioxus [55] and mice [56]. In planarians, the activity of the transcription factor GATA4/5/6 is essential for the correct regeneration and maintenance of the gut [12,13]. A recent study has also uncovered intestine-enriched transcription factors that specifically regulate regeneration (hedgehog signalling effector gli-1) or maintenance (RREB2) of goblet cell types [14], as well as proposing a modest role for Lhx2/9-1 in maintenance of these planarian cell types [14]. Here we further characterize Lhx2/9-1 as a major regulator of goblet cell maintenance and regeneration. Even though we identified the expression of *lhx1/5-2* and *lhx2/9-1* in specific and different gut cell subtypes, we observed that the silencing of either of them strongly reduces the expression of markers for both goblet and basal cells. We also observed reduced expression of phagocyte markers when silencing *lhx1/5-2*. Further experiments might help to analyse whether these effects of the silencing of *lhx1/5-2* on different gut cell subtypes could be explained by its hypothetical expression in a presumptive common gut progenitor of the three main cell lineages. In any case, our results on the function of Lhx1/5-2 and Lhx2/9-1 on intestinal cells expands the limited current knowledge on the function of intestine-rich transcription factors in the regeneration of the planarian gut (figure 7*b*).

Amputation triggers planarian stem cell proliferation and promotes regeneration [97]. Our results suggest that *lhx2/9-3, lhx3/4, lmx1a/b-2* and *lmx1a/b-3* limit the proliferation of the planarian neoblasts. Interestingly, all these genes are in part expressed by planarian stem cells and progenitor cell types [37,64,98], suggesting that they may play a cell autonomous function in controlling stem cell proliferation. These results agree with the reported role for *Lhx5* in the regulation of neural-precursor cell proliferation and migration during formation of the hippocampus in the mouse embryo [99]. In this model, precursor cells for the hippocampal anlagen are specified and proliferate in the absence of *Lhx5*, but many fail to exit the cell cycle (reviewed in [45]). Interestingly, the increased mitotic rates observed in *lhx2/9-3* or *lmx1a/b-2* silenced planarians are associated with regeneration of smaller brains, which could be caused by failure in exiting the cell cycle and in cell differentiation.

Finally, and in relation to the different abnormal phenotypes observed after silencing the several planarian *lhx* genes, it is worth mentioning the role of planarian *islet* genes in axial patterning (figure 7*c*). As previously reported [28,43], we observed that planarian Islet1 is required for the expression of *wnt1* in posterior wounds. Also, it was previously shown that expression of *slit* at the midline of anterior and posterior blastemas at early stages of regeneration depends on *islet1* and it is necessary for correct establishment of the medio-lateral pattern [28]. In agreement, we observed that *islet1* is required for proper expression of *slit* all along the planarian midline during lateral regeneration, suggesting that the observed reduced expression of *slit* could account for the incomplete lateral regeneration and the midline defects observed in *islet1* silenced planarians [72]. In addition, here, we report that a second islet gene, *Islet2*, is expressed in the most anterior tip of the planarian head and is necessary for the stereotypical expression of *notum*. It is known that *notum* and *islet2* are co-expressed in some muscle cells at the most anterior tip of the head region [60]. These *notum* expressing cells, indeed, are absent in *islet2* silenced planarians, which suggests that the expression of *notum* in this domain depends on Islet2. In addition, we observed that *islet2* silenced planarians showed a non-stereotypical expression of the *notum* expressing cells associated to the anterior brain commissure and the eye photoreceptors [61,86], which could be at the origin of the aberrant projections of the visual axons and the elongated brain and head regions observed in *islet2* silenced planarians. However, whether *islet2* and *notum* are co-expressed by these cells still needs to be investigated. Wnt11-6 has a major role in regulating posterior brain growth in planarians [86]. It has been reported that *wnt11-6* silencing produces planarians with bigger brains, a phenotype that could resemble the elongated brains observed in *islet2* silenced planarians. Interestingly, we could identify cells co-expressing *islet2* and *wnt11-6* in the available SC-RNAseq databases. Further experiments will help to elucidate if both genes work together regulating brain size and shape. Finally, in addition to the presence of a larger head-to-body ratio, we observed that the expression of genes such as *ndl-3*, which is known to be required for maintaining normal trunk patterning and regionalized gene expression [89], is strongly reduced in the trunk region and appear ectopically expressed in the pharynx of *islet2* silenced planarians. Also, expression of *ndl-2* and *ndl-5* in the head region decreased in those animals. Thus, altogether we suggest that Islet2 is required to regenerate correct head-to-body proportions. This function might relate to regulation of proper expression of *notum* at anterior ends and *ndl-3* in the trunk region. Further studies should help to elucidate the relationship of *Islet2*, *notum* and *ndl* genes on establishing anterior polarity and proper body proportions.

In other model systems, certain LIM-HDs function in combination in a well-defined ‘LIM code’. Thus, in the mouse developing forebrain, the differentiation of GABAergic neurons and cholinergic neurons is regulated by combinations of Lhx6, Lhx8, and Isl1 (reviewed in [100]); also, in the midbrain, cooperation of Lmx1a and Lmx1b regulates proliferation, specification, and differentiation of dopaminergic progenitors [101]. Similarly, the Lhx1/5 genes *lin-11* and *mec-3* are both required for the terminal differentiation of a subset of specific motor neurons and interneurons in *C. elegans* (reviewed in [45]). In contrast to most invertebrate species that possess six *lhx* genes, one for each of the main LIM-HD subfamilies, we have identified several representatives of the *islet*, *lhx1/5*, *lhx2/9* and *lmx1a/b* classes. These genes have probably originated by internal duplications within the planarian lineage, as has been already reported for other gene families [102–107]. Considering the established combinatorial role of LIM-HD genes in the control of neural identity during embryonic development, the coincident expression of some planarian *lhx* in some neural domains allows us to speculate that they might play a homologous role in specifying combinatorially the neural identify during regeneration of these animals. This is the case, for instance, of the domains of expression of *lmx1a/b-2* and *lmx1a/b-3*, *lhx1/5-1* and *lhx6/8*, or *lhx2/9-2* and *lhx2/9-3*. This hypothesis predicts that some *Smed-lhx* genes should be co-expressed in the same neuronal cells. In support of this hypothesis, our analysis of available SC data suggests a higher-than-expected number of cells co-expressing a given combination of *lhx* genes in the planarian. Moreover, we have visualized by double fluorescence *in situ* hybridization the co-expression for some *lhx* genes, such as for *lhx2/9-3* and *lhx2/9-2* as well as for *lhx2/9-3* and *islet-1*, suggesting that other *lhx* genes might be also expressed in combinatorial patterns, which needs to be further investigated. Alternatively to this hypothesis, the presence of a larger *lhx* repertoire in planarians could have also allowed to redefine specific functions for each of the LIM-HD in specifying distinct and unique cell types. Notably, unlike vertebrates, we have not observed a role for any of the planarian Lmx1a/b paralogues in specifying the dopaminergic neural subtype. We have identified, in contrast, that regeneration of these neurons depends on Islet1. Similarly, both *lhx2/9-2* and *lhx2/9-3* are expressed in lateral domains of the planarian brain, and *lhx1/5-1* and *lhx6/8* cells are detected in the medial region of the cephalic ganglia. Based on the coincidence of the territory of expression of these *lhx* genes in planarians, we cannot discard that the silencing of one of them is counteracted by the expression of the other paralogue/paralogues. Combined RNAi would help to clarify, for instance, if like the mouse counterparts, planarian Lmx1a/b genes work together to regulate dopaminergic neurons or if only *islet1* plays this role. These experiments would identify possible compensatory effects for those genes and further validate the presence of a LIM code in the worm.

In summary, here we report the full repertoire of *lhx* genes in planarians and describe their expression patterns at the levels of whole mount and single cell. RNAi functional analyses have uncovered novel functions for some of these genes mainly in the regeneration of specific neuronal and intestinal cell subtypes as well as in the proper patterning of the brain and body proportions.

## 4. Material and methods

### 4.1. Animal culture

An asexual clonal line of the planarian species *Schmidtea mediterranea* was used for all experiments. Animals were maintained at 18–20°C in artificial planarian water as previously described [108] and fed once a week with organic veal liver. All planarians were starved for at least one week before experiments.

### 4.2. Identification and isolation of *lim* *homeobox* genes

*lim homeobox* genes were identified from the genome of the asexual strain of *S. mediterranea* [109] using the blast tool of Planmine v3.0 [110]. The chromosomic distribution of lhx genes was recovered from the genome assembly at the chromosome level of the sexual strain of *S. mediterranea* (https://simrbase.stowers.org/organism/Schmidtea/mediterranea-Sexual) [111]. The protein sequences of LIM-HD protein homologues of humans, flies, and planarians were used as queries. The protein domain conservation of the planarian candidate transcripts was analysed using the SMART (http://smart.embl-heidelberg.de) and Pfam protein domain databases (http://pfam.xfam.org/). The presence of two LIM domains in the amino termini and a centrally located HD was used as a condition to select the candidates. TRIzol® reagent was used to extract total RNA from a mix of regenerating and intact planarians, and cDNA was synthesized with Superscript III® following manufacturer's instructions. All identified *lim homeobox* genes were amplified using specific primers (electronic supplementary material, file S1). PCR products were cloned into PCRII vectors for synthesis of ssRNA-DIG labelled probes. Synthesis of dsRNA for RNA interference experiments was performed by incorporating T7 and SP6 sequences to the PCR products.

### 4.3. Phylogenetic analyses

Protein sequences of LIM-HD homologues were obtained from NCBI and Planmine v3.0 [110] and aligned using MAFFT with the L- INS-i strategy [112]. The aligned full sequence was used to reconstruct the phylogenetic tree. The phylogenetic tree was inferred with the IQ-TREE Web server, with default options, including the automatic substitution model selector, the ultrafast bootstrap analysis (1000 replicates) and the single branch test number (1000 replicates) [113,114]. The approximate Bayes test option was selected. The phylogenetic tree was visualized using iTOL (https://itol.embl.de) and edited with Adobe Illustrator. Accession numbers for planarian *Schmidtea mediterranea* transcripts retrieved from Planmine can be found in electronic supplementary material, file S1. Accession numbers of LIM-HD protein sequences retrieved from NCBI are as follows: *Drosophila melanogaster* (*Dme*) Lim1A NP_572505.1, Lim3 NP_001260559.1, Lmx1a NP_729801.1, Dme_CG4328 NP_648567.2, Apterous NP_001163058.1, Arrowhead NP_001261379.1, Tailup NP_476774.1; *Homo sapiens* (Hsa) Islet1 NP_002193.2, Islet2 EAW99220.1, Lhx1 NP_005559.2, Lhx2 NP_004780.3, Lhx3 AAG10399.1, Lhx4 NP_203129.1, Lhx5 NP_071758.1, Lhx6 NP_055183.2, Lhx8 AAH40321.1, Lhx9 NP_064589.2, Lmx1b NP_796372.1, Lmx1b AAI43802.1; *Mus musculus* (*Mmu*) Islet1 EDL18368.1, Islet2 EDL25854.1, Lhx1 NP_032524.1, Lhx2 NP_034840.1, Lhx3 AAI50690.1, Lhx4 NP_034842.2, Lhx5 NP_032525.1, Lhx6 CAA04011.1, Lhx8 NP_034843.2, Lhx9 NP_001036042.1, Lmx1a XP_030097992.1, Lmx1b XP_006497809.1; *Nematostella vectensis* (Nve) Islet1 XP_032218609.1, lhx1 XP_001631723.2, lhx3/4 XP_048587815.1, Awh XP_032229933.1, lhx2/9 XP_032240842.1, lmx1 XP_048587096.1; *Strongylocentrotus purpuratus* (Spu) Islet1 XP_781774.3, Lim1 NP_999810.1, Lim1b XP_030853804.1, Lhx2/9 XP_030853437.1, Lhx2/9-like XP_030853097.1, Lhx3/4 XP_030852869.1, Lhx6/8_Awh XP_030853744.1.

### 4.4. Whole-mount *in situ* hybridization

Colorimetric (WISH) and fluorescent (WFISH) whole-mount *in situ* hybridization [115] was performed as previously described [115,116]. Animals were sacrificed by immersion in 5% *N*-acetyl-L-cysteine (5 min), fixed with 4% formaldehyde (15 min) and permeabilized with Reduction Solution (10 min). Riboprobes were synthesized using the DIG- or Fluorescein labelling kits (Sp6/T7) from Roche. Animals were mounted in 70% glycerol before imaging.

### 4.5. Single-cell and bulk RNAseq expression data

The heat map for *lhx* gene expression comparison in the transcriptomes of X1 (neoblasts), X2 (progenitors) and Xins (differentiated) cells according to data in Labbé *et al.* [117] and the heat maps for *lhx* gene expression during regeneration according to data from Wurtzel *et al.* [118] were obtained from https://radiant.wi.mit.edu/app/ [118]. The single-cell sequencing data expression profiles of *lhx* genes during cell differentiation [64] and in neoblasts [98] were obtained from Planmine [110]. Data shown in electronic supplementary material, file S1, about the number of cells expressing a given gene were obtained from the Gene Co-expression Counts tool of PlanEXP found in PlanNET [119,120] using single cell data from Fincher *et al*. [63]. This observed number of cells was compared to the number of cells expected to express a given combination of genes. This number was calculated according to the total number of cells sequenced and the number of cells expressing each of the genes being analysed. We considered that a difference bigger than 10 cells could be an indication of gene co-expression.

### 4.6. RNA interference

Double-stranded RNA (dsRNA) was synthesized by *in vitro* transcription (Sp6/T7 from Roche) as previously described [121]. Two rounds of RNAi were performed for all *lim homeobox* genes. In each round, dsRNA was injected into the digestive system of each planarian during three consecutive days. Three doses of 32.2 nl (at 1 mg ml^−1^) were delivered each day using a Nanoject II (Drummond Scientific Broomall, PA, USA). To induce anterior–posterior regeneration, the head and tail of the animal were amputated on the fourth day. Planarian trunk pieces were allowed to regenerate for 4 days before starting the second round of dsRNA injection and amputation. In the experiments involving sagittal regeneration, planarians were only amputated along their midline after the second round of dsRNA injection. Control animals were injected with *gfp* dsRNA. Regeneration of the treated animals was observed for 10–14 days before proceeding for WISH and immunohistochemistry experiments.

### 4.7. Immunohistochemistry staining

Whole-mount immunohistochemistry was performed as previously described [102,122]. The following antibodies were used: mouse anti-SYNAPSIN, used as pan-neural marker (anti-SYNORF1, Developmental Studies Hybridoma Bank, Iowa City, IA, USA) diluted 1 : 50; mouse anti-VC1 [70], specific for planarian photosensitive cells (anti-arrestin, kindly provided by H. Orii and Professor K. Watanabe) diluted 1 : 15 000; rabbit anti-phospho-histone H3 (Ser10) to detect cells at the G2/M phase of cell cycle (H3P, Cell Signaling Technology) diluted 1 : 300; anti-RAPUNZEL-1 [78], used as a marker for intestinal goblet cells (RPZ-1, kindly provided by K. Bartscherer) used at 1 : 200. The secondary antibodies Alexa 488-conjugated goat anti-mouse and Alexa-568-conjugated goat anti-rabbit (Molecular Probes, Waltham, MA, USA) were diluted 1 : 400 and 1 : 1000, respectively. Samples were mounted in 70% glycerol before imaging.

### 4.8. Planarian feeding behaviour and phototaxis assays

Planarian behaviour to the presence of food was recorded for 15 min using an overhead digital video camera (Canon EOS550D). Phototaxis assay was carried out using a simplified version of the method described by [123]. Planarian behaviour was recorded for 180 s using an overhead digital video camera (Canon EOS550D). To obtain a light gradient, the container was protected by a black screen with a hole that allowed the entrance of 500 lux of white light from one side of the container.

### 4.9. Microscopy, image processing and quantification

Live animals were photographed with an sCM EX-3 high end digital microscope camera (DC.3000s, Visual Inspection Technology) or a digital video camera (Canon EOS550D). Fixed and stained animals were observed with a Leica MZ16F stereomicroscope and imaged with a ProgRes C3 camera (Jenoptik, Jena, TH, Germany). Confocal images were obtained with a Zeiss LSM 880 confocal microscope (Zeiss, Oberkochen, Germany). Image processing and quantifications were performed with Adobe Photoshop and ImageJ2. Counting of the H3P-positive (electronic supplementary material, figure S7) and RPZ-1 positive cells (figure 5) was carried out manually and normalized by the total body area. Brain (figure 2*b*) and head (figure 6) areas were measured in anti-SYNORF1 and DAPI stained planarians and normalized by the total body area. Brain length (figure 6) was measured in anti-SYNORF1 stained planarians and normalized by body length. Signal intensity quantification of colorimetric WISH (figures 5 and 6) was done as previously described [124]: in all experimental conditions compared, animals were developed, stopped, and processed in parallel and for the same time; each single animal was photographed at the same magnification and exposition, and similarly processed with ImageJ2; images were converted in greyscale mode and a threshold was set manually (the same for all images of a given marker); the mean grey value was measured for each image and normalized to the area of the animal; values in graphs are represented as % with respect to the mean of control *gfp*(RNAi) animals.

### 4.10. Statistical analyses and graphical representation

Statistical analyses and graphical representations were realized using GraphPad Prism 9. A box plot displaying the minimum, lower first quartile, median, upper third quartile, and maximum values is used to represent the data. One-way ANOVA was performed to compare the means between conditions after confirming data normality and homogeneity using the Shapiro–Wilk test.

## Data Availability

The data are provided in electronic supplementary material [125].
